# Testosterone Replacement Therapy and the Risk of Hypoglycemia

**DOI:** 10.1155/2019/9616125

**Published:** 2019-11-17

**Authors:** Analía Verónica Yamaguchi, Pablo René Costanzo, Verónica Andrea Peuchot, Pablo Knoblovits

**Affiliations:** ^1^Clinical Medicine Department, Metabolism and Nuclear Medicine, Hospital Italiano de Buenos Aires, Buenos Aires, Argentina; ^2^Department of Endocrinology, Metabolism and Nuclear Medicine, Hospital Italiano de Buenos Aires, Buenos Aires, Argentina

## Abstract

We report the case of a 45-year-old man with a history of Klinefelter syndrome undergoing testosterone replacement therapy, and with type 2 diabetes treated with metformin with poor metabolic control. When vildagliptin was added to his treatment, he presented hypoglycemia after the testosterone injection. We highlight this not widely reported drug interaction between hypoglycemic agents and testosterone.

## 1. Introduction

The use of testosterone as replacement therapy in patients with hypogonadism induces metabolic changes that lead to higher insulin sensitivity (decreased fat mass and increased lean mass, less insulin resistance and higher insulin secretion) [1, 2]. In addition, there is an increased glucose uptake by muscle cells [3]. This effect of testosterone therapy may be associated with a higher risk of hypoglycemia in patients with type 2 diabetes (DM2) under treatment. The interaction of testosterone with insulin has been frequently reported, while little is known about the interaction between testosterone and oral hypoglycemic agents when these drugs are concomitantly administered in hypogonadal men with DM2 [4].

A drug–drug interaction is defined as a change in the effect of a drug by the action of another drug when both are administered together. This action may be synergistic (when the effect is enhanced) or antagonistic (when the effect is decreased). An interaction is considered clinically relevant when the therapeutic activity and/or toxicity of a drug is changed to such an extent that a dosage adjustment of the drug or any other medical intervention is required because of the adverse reactions or significant lack of efficacy caused by such interaction [5, 6]. It should be noted that drug–drug interactions account for 2-3% of hospital admissions in patients older than 50 years of age who are taking medication [7, 8]. The likelihood of a relevant interaction is 50% in patients receiving 5 medications, increasing to 100% in patients receiving 7 drugs simultaneously [9]. In addition, it is important to highlight that oral hypoglycemic agents are a group of drugs with high potential for drug–drug interaction, since they have a narrow therapeutic window; i.e., the therapeutic dose is close to the toxic dose; these drug–drug interactions account for 3.3% of hospitalizations for hypoglycemia [10].

The aim of the publication of this case is to report the risk of hypoglycemia in hypogonadal diabetic patients treated with oral hypoglycemic agents and testosterone.

## 2. Case Report

A forty-five-year-old male patient presented to the clinical medicine department at Hospital Italiano with a 1-year history of DM2 with no medical controls since diagnosis. Personal history: Klinefelter syndrome (KS) diagnosed 4 years ago when the patient consulted for infertility (karyotype 47XXY), with no hormonal replacement, DM2, hypertension (HTN), dyslipidemia (DLD), obesity, sleep apnea treated with continuous positive airway pressure (CPAP). Usual medication: Metformin 1000 mg/day, enalapril 10 mg/day. Physical examination: weight: 110 kg, height: 1.70 m, body mass index (BMI): 38.1 kg/m^2^, sagittal index: 27 cm. Ancillary tests: Labs: blood glucose 89 mg/dL, glycosylated hemoglobin A1c (HbA1c) 8.5%, urea 30 mg/dL, creatinine 0.7 mg/dL, GGT 60 IU/L, total cholesterol 210 mg/dL, triglycerides 173 mg/dL, no-HDL-C 172 mg/dl, LDL-C 137 mg/dL, HDL-C 38 mg/dL, TSH 1.85 *µ*IU/mL (normal: 0.40–4.94 *µ*IU/mL), LH 31.7 mIU/mL (normal: 1.5–9.2 mIU/mL), FSH 26.5 mIU/mL (normal: 1.0–14.0 mIU/mL), prolactin 9.78 ng/mL (normal: 3.0–17.0 ng/mL), estradiol 31.3 pg/mL (normal: 30.0–90.0 pg/mL), total testosterone (TT) 0.79 ng/mL (normal: 3.0–8.8 ng/mL), bioavailable testosterone (BT) 0.21 ng/mL (normal: 1.20–4.80 ng/mL). Testicular ultrasound: both testes of normal shape, with diffuse hypoechogenicity and markedly reduced volume. Ambulatory blood pressure monitoring (ABPM): adequately controlled HTN. Diagnoses: DM2 with poor metabolic control, grade 2 obesity, hypergonadotropic hypogonadism, HTN, DLD, sleep apnea–hypopnea. During the medical interview, the patient reported decreased libido and erectile dysfunction. He was prescribed replacement therapy with testosterone undecanoate (TU) 1000 mg every 12 weeks (the patient was no longer seeking fertility), metformin 1500 mg/day, vildagliptin 100 mg/day and rosuvastatin 10 mg/day.

Forty-eight hours after the administration of the first intramuscular dose of TU 1000 mg, the patient reported symptoms of hypoglycemia (HG) (obnubilation, dizziness, chills), which was not objectively confirmed. At 3 months, the patient complained of the same symptoms 48 hours after the second injection of TU 1000 mg; on this occasion, HG was confirmed by capillary blood glucose levels of 50 mg/dL measured with the Accu-chek Performa® blood glucose meter. Given the low frequency of hypoglycemic events reported with the use of metformin and vildagliptin and the close time relation between the occurrence of clinical symptoms and the intramuscular administration of testosterone, such symptoms were interpreted as a potential drug–drug interaction between testosterone and vildagliptin, since metformin, unlike other oral hypoglycemic agents, does not stimulate insulin secretion, and metformin overdose does not cause hypoglycemia [11]. The patient had no other HG record of capillary blood glucose measurements or laboratory tests.

**Figure 1:**
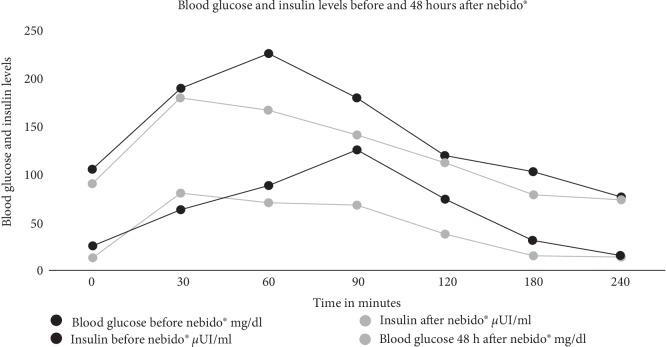
Results of prolonged 75-g OGTT with measurements at timepoints before and after TU administration.

At nine months of clinical follow-up (before the third dose of TU) the patient had good adherence to treatment. Physical examination: weight 95.5 kg (13% loss from baseline weight), BMI: 33.0 kg/m^2^ and good metabolic control: total cholesterol 121 mg/dL, HDL-C 34 mg/dL, triglycerides 104 mg/dL, LDL-C 66 mg/dL, blood glucose 98 mg/dL, GGT 36 IU/L, HbA1c 5.6%, TT 2.15 ng/mL, BT 0.7 ng/mL (15 weeks after the last injection of TU), PSA 0.54 ng/mL, 24-h microalbuminuria: 248.8 mg/24 h (normal: 0–30.0 mg/24 h). Vildagliptin was discontinued and the patient was asked to undergo a prolonged oral glucose tolerance test (OGTT) with blood glucose measurements at various timepoints, prior to and 48 hours after the next administration of testosterone. (Table 1, Figure 1).

The patient made good progress; 3 months after discontinuation of vildagliptin, a new dose of testosterone undecanoate was administered and the patient had no symptoms of HG. Follow-up labs were: blood glucose 88 mg/dL, HbA1c 5.5%, TT 2.5 ng/mL, BT 1.2 ng/mL, SHBG 27 mMol/L.

## 3. Methods

Blood samples were taken at 8:00 AM after 12-hour nocturnal fasting.

 
*Insulin*: Chemiluminescence (Architect I2000-Abbott). Reference range: 2.7–10.4 *μ*IU/mL. Analytical sensitivity: <1.0 *μ*IU/mL, with intra-assay and inter-assay coefficient of variation (CV) below 7.0%.
 
*Total Testosterone*: Chemiluminescence on the Immulite 2000 (Siemens) analyzer, laboratory reference ranges: 1.3–8.5 ng/mL for adult males; the intra-assay CV was 9.8% and the inter-assay CV was 14.4%.
 
*Bioavailable Testosterone*: Vermeulen calculation.
 
*Glucose*: UV enzymatic hexokinase method (AU5800 Beckman coulter).
 
*OGTT*: The patient arrived at the laboratory between 7 AM and 8.30 AM in an 8-hour fasting state. A baseline sample was collected after the 8-hour fast for blood glucose and insulin measurement, and the patient was asked to drink 75 g of glucose in 375 ml of water independent of his baseline fasting glucose levels. Samples were collected for blood glucose and insulin measurements at 30, 60, 90, 120, 180 and 240 min after intake, according to the World Health Organization criteria [12].

Pharmacokinetics and pharmacodynamics of the drugs used by this patient.

*Testosterone Undecanoate*: Pharmacodynamic properties: TU is an ester of the naturally occurring androgen, testosterone. The active form is formed by cleavage of the side chain. Pharmacokinetic properties:* Absorption: *TU is administered intramuscularly, thus avoiding the first-pass effect of the liver. Following intramuscular injection, it is gradually released from the depot and almost completely cleaved by serum esterases into testosterone and undecanoic acid. An increase in serum testosterone concentrations above basal values may be measured one day after administration. *Distribution: *In two separate studies, mean maximum concentrations of testosterone of 6.92 and 12.97 ng/mL were measured about 7 and 14 days, respectively, after single IM administration of 1000 mg of testosterone undecanoate to hypogonadal men. Post-maximum testosterone concentrations decreased with an estimated half-life of approximately 53 days. *Metabolism: *Testosterone generated by ester cleavage from testosterone undecanoate is metabolized and excreted in the same way as endogenous testosterone (extensive hepatic and extrahepatic metabolism). The undecanoic acid is metabolized by *β*-oxidation in the same way as other aliphatic carboxylic acids.

Vildagliptin is a dipeptidyl-peptidase-4 (DPP-4) inhibitor. This inhibitory activity results in an increase in the levels of incretin hormones, among them GLP-1 (glucagon-like peptide 1) and GIP (glucose-dependent insulinotropic peptide), which stimulates insulin secretion and reduces glucagon secretion in a glucose-dependent manner [13]. Hypoglycemia does not appear to be a serious problem when vildagliptin is used as monotherapy (0.4% of patients treated with 100 mg/day of vildagliptin compared to 0.2% of patients treated with an active comparator or placebo) or as dual therapy in combination with metformin versus placebo (0.55% vs. 0.54%, respectively) or with pioglitazone versus placebo (0.3% vs. 1.9%, respectively). No serious hypoglycemic events were reported with vildagliptin therapy [14]. Interactions: Vildagliptin has a low potential for interactions with other drugs. Since vildagliptin is not a cytochrome P (CYP) 450 enzyme substrate, does not inhibit or induce CYP 450 enzymes, and is a weak substrate of *P*-glycoprotein, no relevant pharmacokinetic interactions have been observed after the concomitant administration of vildagliptin with pioglitazone, metformin, glyburide, amlodipine, ramipril, valsartan and simvastatin in healthy subjects.

*Metformin*: Unlike sulfonylureas, metformin does not stimulate insulin secretion; therefore it is more appropriate to refer to it as an insulin-sensitizing agent or antidiabetic drug. In overdose, metformin does not cause hypoglycemia.

## 4. Discussion

Men with KS have an increased mortality risk, probably due to their higher predisposition to develop congenital malformations, cardiovascular disease and endocrine-metabolic disorders, compared to age-matched controls [15].

Our patient had several cardiovascular risk factors, including HTN, DM2, DLD and obesity, the most commonly reported comorbidities in patients with this type of karyotypic abnormality. The prevalence of metabolic disorders in men with KS is associated with insulin resistance and DM2, and with an increased mortality from these causes [16]. Furthermore, hypogonadism per se is a risk factor for metabolic disorders and cardiovascular disease.

Patients with KS have a high prevalence of hypogonadism due to Leydig cell dysfunction, but in addition, metabolic abnormalities in this patient may contribute to a decline in testosterone levels. Insulin resistance-associated hyperglycemia (or hyperinsulinemia) contributes to a lower hypothalamic activity with a decrease in the number of gonadotropin-releasing factor pulses [17, 18]. However, other studies have shown a decreased testicular response to chorionic gonadotropin stimulation in patients with insulin resistance (IR), evidencing testicular function impairment in metabolic disorders [19]. In support of these findings, it has been demonstrated that in the presence of obesity and IR, Leydig cell steroidogenesis may be impaired due to IR at this level or to the action of hormones or cytokines from visceral fat [20–22].

Since hypogonadism in KS is a hormone deficiency of primary (testicular) origin, it could be stated that this condition might have been in our patient the initial manifestation in the development of metabolic disorder, and the development of metabolic disease might have subsequently further impaired testosterone production by mechanisms directly affecting the testicles. However, as patients with KS are, per se, more prone to the development of metabolic syndrome independently of their testosterone levels, it is highly likely that in our patient both syndromes might have influenced each other in a positive feedback manner, leading to the current medical condition.

Testosterone replacement therapy in hypogonadal patients leads to changes in body composition with a decrease in fat mass and an increase in lean mass, and it induces changes in insulin sensitivity [1]. After replacement therapy, there is a decline in insulin secretion and less IR, and an increase in the expression of the glucose transporter Glut4 and enhancement of its translocation, resulting in increased glucose uptake by muscle cells [2, 3]. These changes determine that long-term treatment with testosterone is associated with weight loss, improves glycemia, lipids and prevents the evolution of prediabetes to DM2 [23, 24]. In addition, complete remission of DM2, risk reduction of cardiovascular disease and mortality was reported with TU treatment [25–28].

Thus, in this case, in order to evaluate the insulin-sensitizing response of testosterone, a prolonged OGTT was performed with glucose and insulin measurements at 0, 30, 60, 120, 180 and 240 minutes before and after the administration of TU. The curve shows an insulin-sensitizing effect after TU administration, with a decrease in blood glucose and insulin levels.

We think that in this case there was no effect of one drug on the other drug's metabolism (vildagliptin and testosterone), but a synergistic action of both in the decrease of blood glucose levels. This explanation is reinforced by the fact that there is not demonstration that testosterone alone is capable of producing HG.

Metabolic disorders, especially DM2 and IR are really frequent in patients with KS [16]. Testosterone replacement or treatment with hypoglycemic agents or insulin is unavoidable, but before the initiation of therapy, it is important to be aware of the fact that androgens may enhance the hypoglycemic effect of blood glucose-lowering agents. Patients should be monitored for hypoglycemia so that, if needed, changes may be made in prescription or doses of hypoglycemic agents when administered in combination with androgens.

Even if for testosterone interactions with insulin have been most frequently reported [4] and little evidence is available on interactions between testosterone and oral hypoglycemic drugs, particularly DPP4 inhibitors, such interactions, especially hypoglycemic events as the case reported in this paper, should not be ruled out. This synergism resulting in hypoglycemia occurred in an acute manner 48 hours after the administration of testosterone and it did not recur when vildagliptin was discontinued. However, after testosterone administration, there was an improvement in basal and post-load levels of insulin and blood glucose. It is important that treating physicians be aware of this potential interaction so that they may adjust the doses of oral hypoglycemic agents, if needed.

## Figures and Tables

**Table 1:** Results of prolonged 75-g OGTT with measurements at timepoints before and after TU administration.

Timepoints	Blood glucose before TU (mg/dL)	Insulin before TU (*µ*IU/mL)	Blood glucose 48 hours after TU (mg/dL)	Insulin after TU (*µ*IU/mL)
Baseline	106	25.6	91	13.8
30 min	190	63.3	180	80.8
60 min	226	88.7	167	71.4
90 min	181	125.6	142	68.5
120 min	120	74.9	113	38.6
180 min	104	31.9	80	16.1
240 min	77	15.7	74	14.3

TU: Testosterone Undecanoate.
